# Parametric Study on Responses of a Self-Anchored Suspension Bridge to Sudden Breakage of a Hanger

**DOI:** 10.1155/2014/512120

**Published:** 2014-06-12

**Authors:** Wenliang Qiu, Meng Jiang, Cailiang Huang

**Affiliations:** School of Civil Engineering, Dalian University of Technology, Dalian 116024, China

## Abstract

The girder of self-anchored suspension bridge is subjected to large compression force applied by main cables. So, serious damage of the girder due to breakage of hangers may cause the collapse of the whole bridge. With the time increasing, the hangers may break suddenly for their resistance capacities decrease due to corrosion. Using nonlinear static and dynamic analysis methods and adopting 3D finite element model, the responses of an actual self-anchored suspension bridge to sudden breakage of hangers are studied in this paper. The results show that the sudden breakage of a hanger causes violent vibration and large changes in internal forces of the bridge. In the process of the vibration, the maximum tension of hanger produced by breakage of a hanger exceeds 2.22 times its initial value, and the reaction forces of the bearings increase by more than 1.86 times the tension of the broken hanger. Based on the actual bridge, the influences of some factors including flexural stiffness of girder, torsion stiffness of girder, flexural stiffness of main cable, weight of girder, weight of main cable, span to sag ratio of main cable, distance of hangers, span length, and breakage time of hanger on the dynamic responses are studied in detail, and the influencing extent of the factors is presented.

## 1. Introduction

Large-span bridges, including cable-stayed bridge, suspension bridge, and arch bridge, need cables to be the stay cables, main cables, or hangers. With the time increasing, the steel elements of bridge are exposed to corrosion, and their resistance capacities decrease. The stay cables and hangers especially are more prone to corrosion than other elements for the diameters of their steel wires are small. Under the combination action of live loads and corrosion, stay cables and hangers may break suddenly. In china, serious corrosion and breakage of cables occurred in many bridges [1]. In 2001, eight hangers of Yibin Southgate Bridge in China broke, and the deck supported by these hangers fell into the river. Two men died in this accident. In 1995, one stay cable of Guangzhou Haiyin Bridge that was only used for 7 years broke, and it hit an oil tank truck. To avoid the breakage of cables due to corrosion, cables in many bridges were replaced in China. For example, all the stays in Jiujiang Bridge that was used for 10 years were replaced, because 70% of the stays were seriously corroded, and 1/3 of wires in some stays were broken.

**Figure 1:**
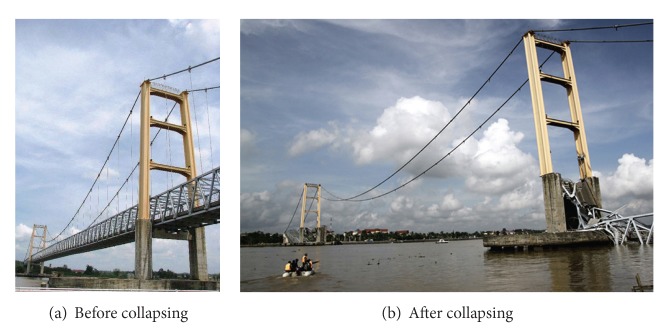
Mahakam II Bridge in Indonesia.

Because the breakage of cable usually occurs suddenly, and it can cause strong vibration and large change of internal forces of the structure, the sudden breakage may endanger the safety of the bridge. Because one hanger is broken, Mahakam II suspension Bridge in Indonesia collapsed in 2011, as shown in Figure 1. It happened very fast, only about 30 seconds. This bridge was completed in 2002, and it was less than 10 years old. At least 11 people were dead and 30 people were missing in this accident.

The effects of breakage of stay cables on cable-stayed bridge have been studied by many researchers [2–8]. Considering the influences of different layout of stays, number of planes of stays, and stiffness of deck, Mozos and Aparicio [5, 6] studied the effects of breakage of stays on deck, tower, and stays in detail. The study showed that it was safe for stays when adopting the dynamic amplification factor (DAF) of 2.0 recommended by PTI [9], but it was unsafe for deck and tower. Because the time that the breakage occurs in (breakage time) has significant effects on the responses of structure, Mozos and Aparicio [10] studied the breakage time through experiments and found that the breakage time was 0.00375 s for damaged cables, and 0.0085 s for undamaged cables. Zhou and Chen [8] studied dynamic responses of a cable stayed bridge caused by abrupt cable-breakage considering dynamic bridge-vehicle interactions.

Considering the geometric and material nonlinearity, Cai et al. [11] studied the nonlinear responses and progressive collapses of cable-stayed bridge due to sudden breakage of stays. When a single stay broke, the tension of other stays changed little. When two adjacent stays broke simultaneously, the bridge collapse did not occur, but the deck had large plastic deformation and the stays yielded. Qu et al. [12] studied DAFs of Sutong Bridge in China and found that the DAFs did not exceed 3%.

Ruiz-Teran and Aparicio [13] studied the effects of breakage of stays on underdeck cable-stayed bridge. The influences of the breakage time and the dynamic loads of breakage were investigated. When the breakage time was less than 10% of the fundamental period of the damaged bridge, it had no influences on the results. The study results showed that the responses produced by breakage of stays must be calculated using dynamic analysis method.

Considering the geometric and material nonlinearity, Qiu et al. [14] and Kao et al. [15] studied the static load-bearing capacity of self-anchored suspension bridge. The studies showed that the bridge did not collapse when five hangers broke. Because the dynamic effects of the breakage of hangers were not considered, the load-bearing capacities obtained by the studies are the upper limit value.

In recent years, more than 20 self-anchored suspension bridges have been built in China [16]. Their hangers are all made of parallel high strength galvanized steel wires. In Chinese codes [17], the safety factor of hanger is 3.0 during service, and it is 1.8 during replacement of hanger. No specifications are presented for sudden loss of hangers. Because the hangers are connected with the main cables, the breakage of hangers will induce the main cable to vibrate strongly, which will further induce strong vibration and large changes of internal forces of the whole bridge. Using nonlinear static and dynamic analysis methods, the responses of a self-anchored suspension bridge due to the breakage of hangers are studied in this paper. The results can be used for reference in design and maintenance of this kind of bridge.

## 2. Structure of a Self-Anchored Suspension Bridge and Analysis Methods

### 2.1. Structure of a Self-Anchored Suspension Bridge

**Figure 2:**
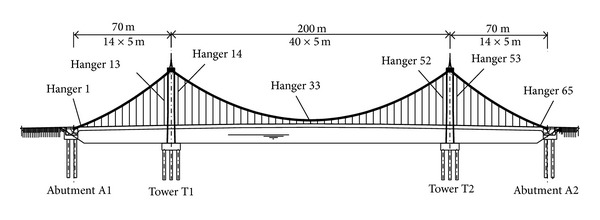
Layout of Zhuanghe Jianshe Bridge.

**Figure 3:**
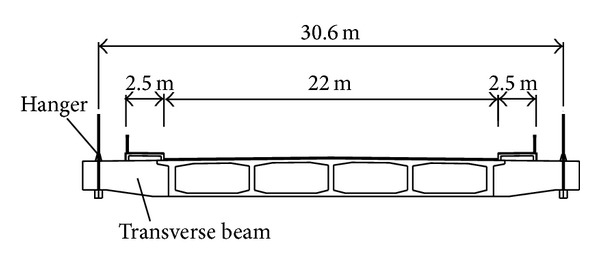
Cross-section of girder.

Zhuanghe Jianshe Bridge built in China is a concrete self-anchored suspension bridge with mid-span of 200 m and side-span of 70 m, as shown in Figure 2. Its stiffening girder with box cross-section is reinforced concrete, as shown in Figure 3. Because the girder is subjected to a large compressive force applied by the main cables and the tension stresses do not occur in the girder under design loads, the tendons are not used in the girder. The tower is reinforced concrete with box cross-section. The bridge has two main cables and each cable is made of 3937 paralleled, 5 mm in diameter, high strength galvanized steel wires. The span to sag ratio of main cable in mid-span is 5.5. There are 65 hangers on each side of the bridge, and the distance of the hangers along the girder is 5 m. The hangers are made of 97 paralleled, 7 mm in diameter, steel wires. The hangers are numbered from 1 to 65.

### 2.2. Materials

The concrete of the girder and the towers is high strength concrete with a compressive strength of 50 MPa and a modulus of elasticity of 34000 MPa. The density of the reinforced concrete is 25.5 kN/m^3^. The steel of the main cables and the hangers has an ultimate tensile strength of 1570 MPa and a modulus of elasticity of 190000 MPa. The density of the steel is 78.5 kN/m^3^.

### 2.3. Loads

Because this study is only used to investigate the responses of bridge caused by sudden breakage of hangers, the analysis of the structure under dead loads is carried out. The self-weight of girder, towers, main cables, and hangers is obtained from multiplying the areas of their cross-sections by their densities. The distributed dead load on the deck except self-weight is 108 kN/m. The distributed dead load on the main cables except self-weight is 0.37 kN/m. Because the weight of clamps affects the vibration of the main cable, it is also considered in dynamic analysis. The weight of each clamp from 1 to 9 is 12 kN, the weight of each clamp from 10 to 17 is 16 kN, and the weight of each clamp from 18 to 33 is 10 kN.

### 2.4. Analysis Model

**Figure 4:**
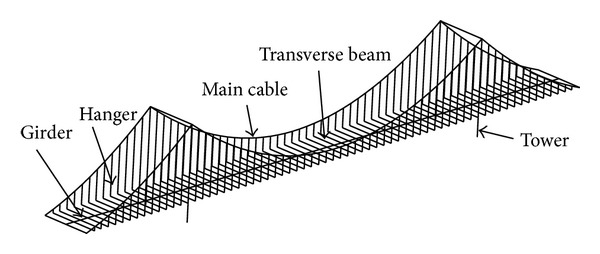
FE model of the bridge.

The bridge is modeled with three-dimensional (3D) finite element (FE) model, as shown in Figure 4. In the FE model, the girder, towers, and transverse beams are modeled using 3D beam elements, and their torsion stiffness and torsion mass are considered. In order to study the influence of flexural stiffness, the main cables are modeled using beam elements. The hangers are modeled using truss elements. The influences of initial internal forces under self-weight on the stiffness of the structure are considered. The main cables between two hangers are modeled using 5 elements to consider geometrical configuration and local vibration of the main cables more precisely. The masses of clamps are concentrated on the nodes where the clamps are located. Considering the piles of the bridge had little effects on the analysis of sudden breakage of hanger, they are not modeled in the FE model. The girder is supported by two bearings at each tower as well as at each abutment. Using this FE model, the modal properties of Zhuanghe Jianshe Bridge are calculated. The lowest natural frequency of the vertical vibration mode of the bridge is 0.41 Hz, and the lowest natural frequency of the torsional vibration mode of the girder coupled with transversal vibration is 1.00 Hz.

### 2.5. Analysis Methods

**Figure 5:**
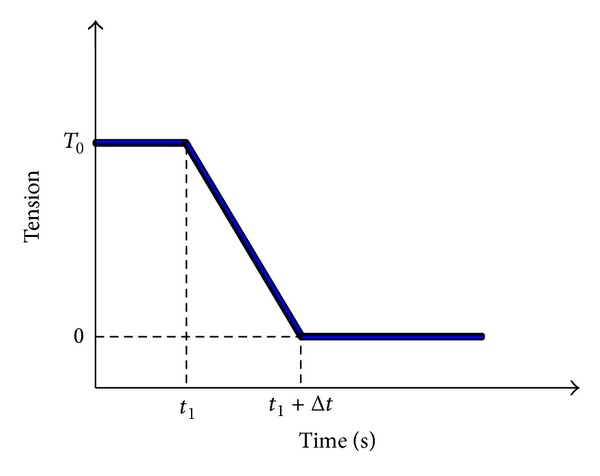
Time history of tension of broken hanger.

Responses of the bridge due to sudden breakage of hangers on the bridge are analyzed by means of nonlinear static and dynamic analysis using the finite element software ABAQUS V.6.8. For either static analysis or dynamic analysis, the geometric nonlinearity of the structure and effects of axial forces of the structure on the stiffness are considered, and the nonlinear procedures are carried out using iteration method. In dynamic analysis, the direct time integration method is used. The time history of the tension of the broken cable is shown in Figure 5. Before the broken hanger breaks, the value of its initial tension is *T*_0_. When the hanger breaks at time *t*_1_, its tension drops from *T*_0_ to zero during time interval* Δt*.

Here,* Δt* is called breakage time of hanger, which takes a value of 0.005 s [5] except when studying the influences of the breakage time on the dynamic responses.

**Figure 6:**
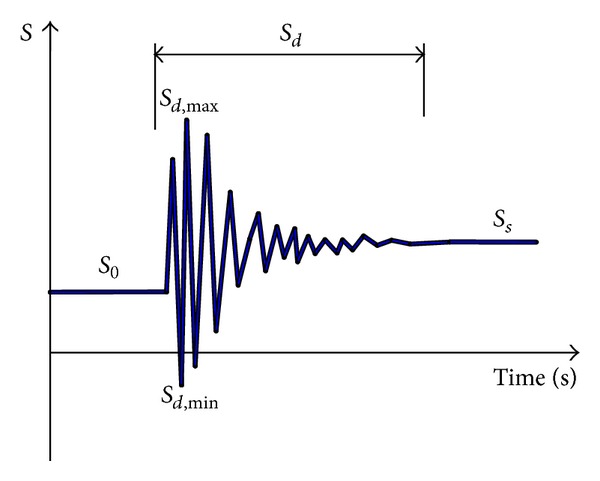
Time history of structural response.

The following analysis processes are adopted for analysis, as shown in Figure 6.

- Using nonlinear static analysis method and adjusting the initial stresses of main cables, hangers, girder, and towers, a reasonable static state of the undamaged bridge under dead loads is reached. In this state, the tensions of all hangers are nearly equal, and the bending moments of the girder and towers are very small.
- A hanger element is removed, and its tension is unloaded in time interval Δ*t*.
- The structure vibrates strongly due to the sudden breakage of the hanger, and the dynamic analysis is carried out to calculate the vibration of the structure. Because of damping, the vibration attenuates with time increasing. Until the maximum node displacement amplitude of the structure is less than 0.1 mm, the state of bridge is taken as the final equilibrium state under dead loads after breakage of a hanger.

The static state before breakage of the hanger is identified by *S*_0_. After the sudden breakage of a hanger, the structure vibrates strongly, and the dynamic state of the bridge is identified by *S*_*d*_. It is especially mentioned that the final static state obtained by dynamic analysis is just the same as the static states of bridge without the broken hanger obtained by static analysis. The final static state after breakage is identified by *S*_*s*_.

After a preliminary numerical study on the dynamic responses of the bridges to pulse loads, the time step of 0.001 s is used to calculate the dynamic process during 10 seconds after the sudden breakage of hanger, and the time step of 0.005 s is used during the other time. The time steps allow us to achieve an important reduction in computing time and to maintain adequate accuracy in the results. A Rayleigh damping of 2% is used in the dynamic analysis.

## 3. Reponses due to Sudden Breakage of a Single Hanger

Considering that two hangers are not possible to break at the same time, the dynamic responses caused by breakage of a single hanger is studied in this paper. From the dynamic analysis results, it can be found that the sudden breakage of a hanger produces very strong vibration of the structure; some internal forces of the structure become very large during the vibration. The bending moments of girder, bending moments of towers, and tensions of main cables caused by the sudden breakage of a hanger are not large enough to control the design. The tensions of hangers and reactions of bearings are so large that they may control the design.

**Figure 7:**
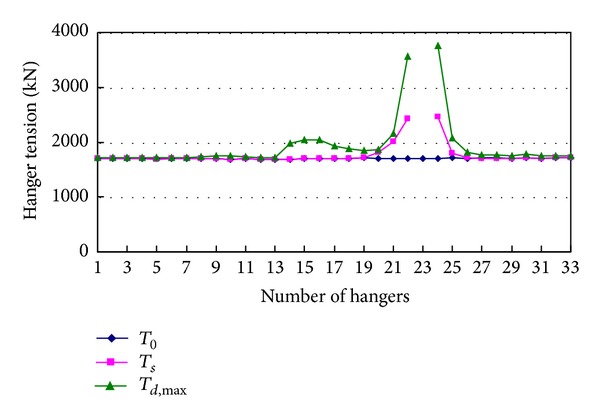
Hanger tensions caused by breakage of hanger 23.

Figure 7 shows the initial value *T*_0_, final value *T*_*s*_, and maximum value *T*_*d*,max⁡_ of tensions of the other hangers when hanger 23 breaks. It can be seen from the figure that breakage of a hanger has large effects on tensions of the hangers near the broken hanger and has little effects on tensions of the hangers far away from the broken hanger. The maximum tension *T*_*d*,max⁡_ of each hanger during vibration is much larger than the corresponding final value *T*_*s*_.

**Figure 8:**
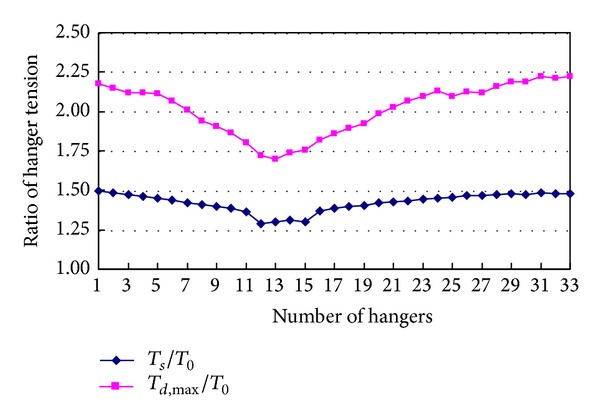
Ratios of hanger tensions.

To obtain the maximum values of tension ratios *T*_*d*,max⁡_/*T*_0_ and *T*_*s*_/*T*_0_ of every hanger, maximum tension *T*_*d*,max⁡_ and final tension *T*_*s*_ of each hanger are calculated when every one of the other hangers breaks.  Figure 8 shows the maximum values of tension ratios *T*_*d*,max⁡_/*T*_0_ and *T*_*s*_/*T*_0_ of the hangers from 1 to 33. The tension ratio *T*_*d*,max⁡_/*T*_0_ of hanger 30 is the largest, that is, 2.22. However, the tension ratio *T*_*s*_/*T*_0_ of hanger 30 is only 1.48, which is only 66.7% of the tension ratio *T*_*d*,max⁡_/*T*_0_. So, it can be concluded that the difference of the results between static analysis and dynamic analysis cannot be neglected. Additionally, the hanger is farther from the tower, the vibration caused by its breakage is stronger. So the tension of hanger increases with the distance between the hanger and tower increasing.

The dynamic effects on hanger tensions are so marked that the maximum tension reaches 2.2 times of the initial value. If the breakage of a hanger is for the reason of corrosion, the other hangers may be also exposed to corrosion, and their resistance capacities decrease too. The breakage of one hanger may induce one-by-one breakage of the other hangers, and the bridge may collapse after several hangers break. So, the breakage of a hanger can endanger the safety of the bridge seriously.

**Figure 9:**
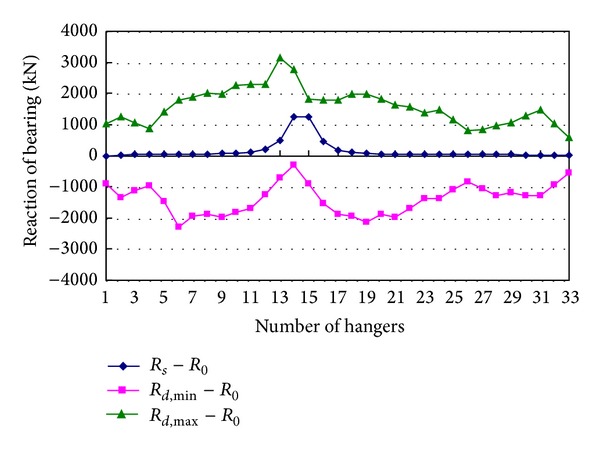
Reaction of the left bearing at tower T1.

**Figure 10:**
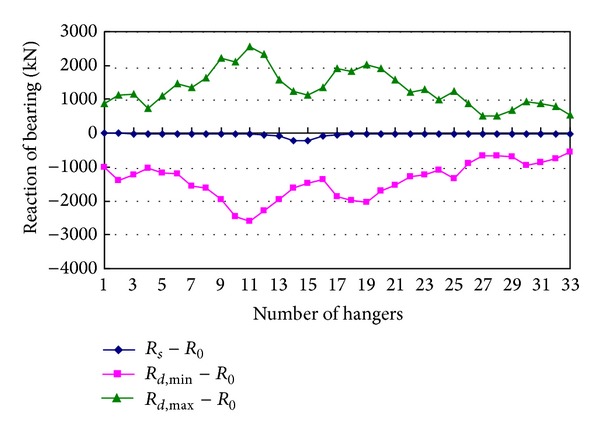
Reaction of the right bearing at tower T1.

The reaction forces *R*_*d*,max⁡_ − *R*_0_, *R*_*d*,min⁡_ − *R*_0_, and *R*_*s*_ − *R*_0_ of the two bearings at tower T1 produced by sudden breakage of every one of the hangers from 1 to 33 on the left side are shown in Figures 9 and 10. Because breakage of any one hanger can induce strong vertical and torsional vibrations, vibrations further induce large changes of reaction forces of bearings. The maximum increment of reaction force of the left bearing is produced by breakage of hanger 13, and it is 1.86 times the tension of the broken hanger. The maximum decrement of reaction force of the right bearing is produced by breakage of hanger 6, and it is 1.35 times the tension of the broken hanger. Both the maximum increment and the maximum decrement of reaction force of the right bearing are produced by breakage of hanger 11, and they are 1.51 and 1.54 times the tension of the broken hanger, respectively. So, the reaction forces of bearings produced by breakage of a hanger cannot be ignored. Additionally, the reaction forces of bearings due to torsion of girder are related to the distance between the two bearings, and the reaction forces increase with the distance decreasing. A relatively large distance is adopted in the bridge analyzed in this paper.

## 4. Parametric Study on Influencing Factors

**Figure 11:**
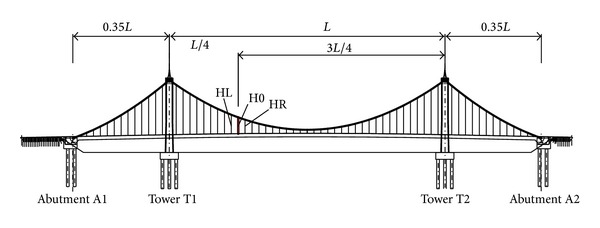
Positions of hangers H0, HL, and HR.

In order to find out the relationship between the responses caused by breakage of hanger and the structure, based on the bridge analyzed above, breakage of the left hanger H0 at the position of 1/4 of mid-span, as shown in Figure 11, is taken as an example to study the influences of flexural stiffness of girder, torsion stiffness of girder, flexural stiffness of main cable, weight of girder, weight of main cable, span to sag ratio of main cable, distance of hangers, span length, and breakage time of hanger on the dynamic responses. The mid-span length is 200 m for all above factors except span length. For general suspension bridge, the initial tensions of all hangers under dead load are designed to be a same value. In the suspension bridge analyzed in this paper, the initial tension of each hanger is *T*_0_. To demonstrate the extent of the influences on hanger tensions, ratio of the maximum tension *T*_*d*,max⁡_ to initial tension *T*_0_ is adopted in the following analysis. Because the breakage of hanger H0 has marked influence only on the adjacent hangers HL and HR, only the tensions of the two adjacent hangers are presented when the influencing factors are studied parametrically. Additionally, considering that the bearing reactions change largely during the dynamic responses, they are presented for a part of the influencing factors.

### 4.1. Flexural Stiffness of Girder

**Figure 12:**
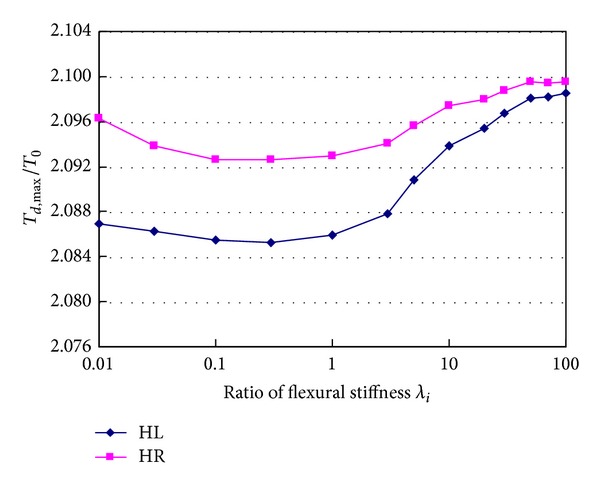
Effects of flexural stiffness of girder on hanger tensions.

**Figure 13:**
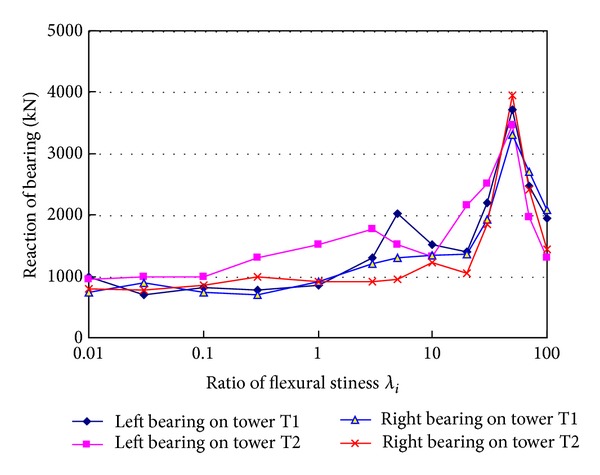
Effects of flexural stiffness of girder on bearing reactions.

Figure 12 shows relationship between tension ratio *T*_*d*,max⁡_/*T*_0_ of hangers HL and HR and flexural stiffness of girder when hanger H0 breaks suddenly. In the figure, *λ*_*i*_ is ratio of the changed flexural stiffness to the actual flexural stiffness of the designed girder. When the flexural stiffness of girder increases from 0.1 to 100 times of actual value, the tension ratio of hanger HL changes in the range of 2.085~2.099, and it changes only by 0.67%. The tension ratio of hanger HR changes in the range of 2.093~2.100, and it changes only by 0.33%. It can be concluded that flexural stiffness of girder has small effects on hanger tension.  Figure 13 shows reactions of the four bearings at the two towers change with the flexural stiffness ratio increasing. It can be seen from the figure that the bearing reactions increase with the flexural stiffness of girder increasing. When the flexural stiffness ratio is between 0.01 and 3.0, the reactions caused by breakage of hanger H0 are nearly same except for the reaction of the left bearing on tower T2. But when the flexural stiffness ratio is larger than 3.0, the bearing reactions change very markedly. Particularly, when the flexural stiffness ratio is about 50.0, the peak values occur in all curves at the same time. The maximum reaction is 3946.5 kN, which is 2.31 times the initial tension of the broken hanger (*T*_0_ = 1705.2 kN). From the vertical vibration of the structure, it can be seen that local vibration of main cable induces resonance of the global bridge when flexural stiffness of girder takes a certain value.

### 4.2. Torsion Stiffness of Girder

**Figure 14:**
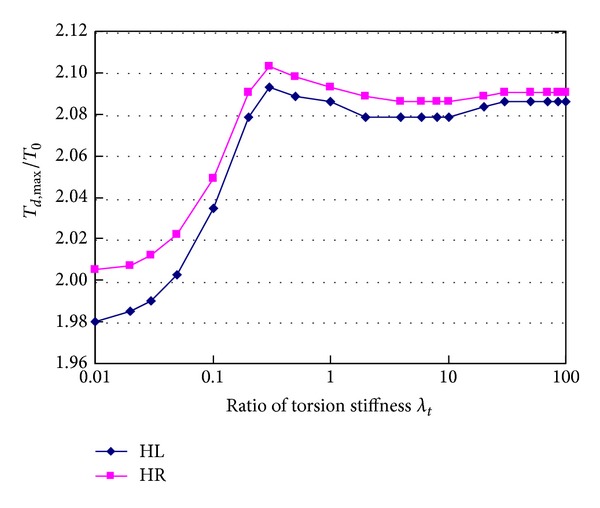
Effects of torsion stiffness of girder on hanger tensions.

**Figure 15:**
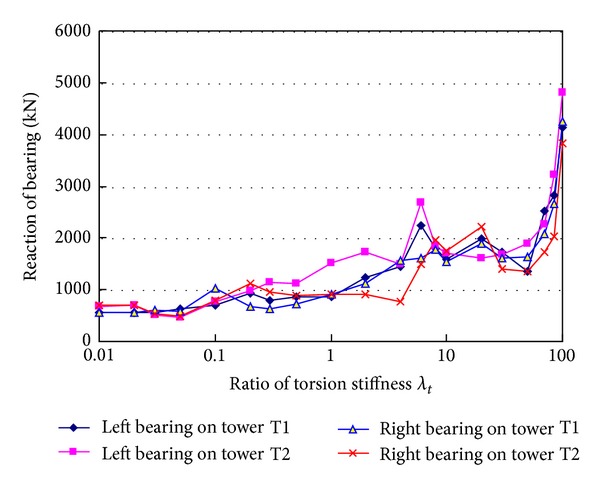
Effects of torsion stiffness of girder on bearing reactions.

Figure 14 shows relationship between tension ratio *T*_*d*,max⁡_/*T*_0_ of hangers HL and HR and torsion stiffness of girder when hanger H0 breaks suddenly. In the figure, *λ*_*t*_ is ratio of the changed torsion stiffness to the actual torsion stiffness of the designed girder. When the torsion stiffness ratio increases from 0.1 to 0.3, the tension ratio of hanger HL increases from 1.980 to 2.093, the tension ratio of hanger HR increases from 2.005 to 2.103, and they increase 5.7% and 4.9%, respectively. When the torsion stiffness ratio increases from 0.3 to 4.0, the hanger tension ratios decrease a bit. When the torsion stiffness ratio is larger than 4.0, the hanger tension ratios remain nearly unchanged.  Figure 15 shows the trend that the bearing reactions increase with the increment of torsion stiffness of girder. When the torsion stiffness ratio increases from 0.01 to about 50, the reactions are nearly in the range of 750~2000 kN. When the torsion stiffness ratio is larger than 50, the reactions increase markedly, and the maximum value is 4814.0 kN at the stiffness ratio of 100.

### 4.3. Flexural Stiffness of Main Cable

**Figure 16:**
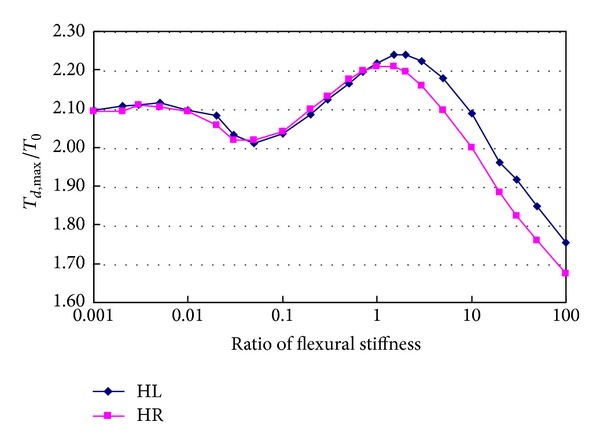
Effects of flexural stiffness of main cable on hanger tension.

Generally, the flexural stiffness of main cable is neglected and the main cables are modeled using cable elements. The above assumption is reasonable when the local vibration of main cable is not considered in structural analysis of suspension bridge. Because the flexural stiffness has large effects on the dynamic characteristics of the cable, it cannot be ignored in analysis of the violent local vibration of main cable caused by sudden breakage of hanger. In this paper, the main cable is assumed as a solid cylinder with curved geometry, which has the same sectional area as the actual main cable. By changing the flexural stiffness of main cable from 0.001 to 100 times the flexural stiffness of the solid cylinder, the effects of the flexural stiffness of main cable on the dynamic responses caused by breakage of hanger H0 are studied.  Figure 16 shows the relationships between tension ratios of hangers HL and HR and the flexural stiffness ratio of main cable. It can be seen that the flexural stiffness of main cable has very marked influences on hanger tensions. When the flexural stiffness ratio of main cable increases from 0.001 to 0.01, the tension ratios nearly remain a constant value of about 2.10. When it increases from 0.01 to 0.05, the tension ratio decreases to be a value of about 2.01. When it increases from 0.05 to 1.5, the tension ratio increases to be a maximum value of 2.240. When it increases from 1.5, the tension ratio decreases rapidly. The tension ratio of hanger HR decreases to be a value of 1.675 when the flexural stiffness ratio is 100.

### 4.4. Weight of Structure

**Figure 17:**
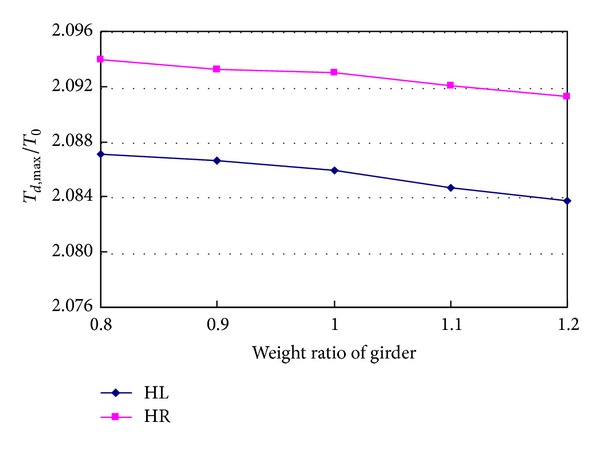
Effects of weight of girder on hanger tension.

Weight of structure is one of the primary factors that affect the dynamic characteristics of the bridge. By changing weight of girder and main cable, the effects of weight on the dynamic responses caused by breakage of hanger H0 are studied.  Figure 17 shows the changes of tension ratios of hangers HL and HR when the weight of girder increases from 0.8 to 1.2 times the actual weight of the designed girder. The tension ratios change less than 0.2%, so the effects of weight of girder can be ignored.

**Figure 18:**
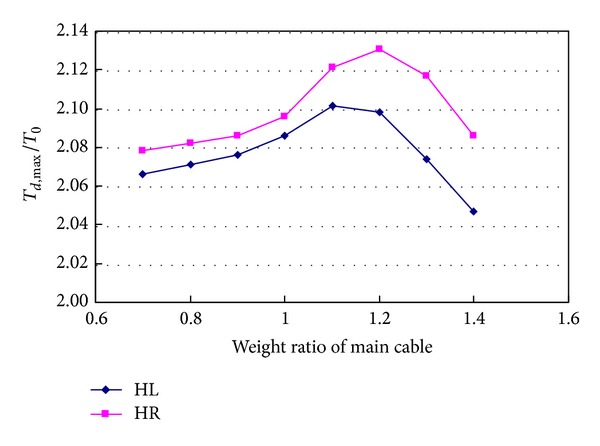
Effects of weight of main cable on hanger tension.

Figure 18 shows the relationship between tension ratios of hanger and the weight of main cable. It can be seen from the figure that the weight of main cable has larger effects than the weight of girder. When the weight of main cable increases from 0.7 to 1.4 times the actual weight of the designed main cable (including the weight of steel wires of cable, clamps, wrapping, and decoration), tension ratio of hanger HL changes in the range of 2.047~2.101, and tension ratio of hanger HR changes in the range of 2.079~2.131. They change by 2.6% and 2.5%, respectively. From Figures 16 and 18, it can be seen that dynamic characteristics of main cable determined by flexural stiffness and weight have marked effects on the tensions of the hangers.

### 4.5. Span to Sag Ratio of Main Cable

**Figure 19:**
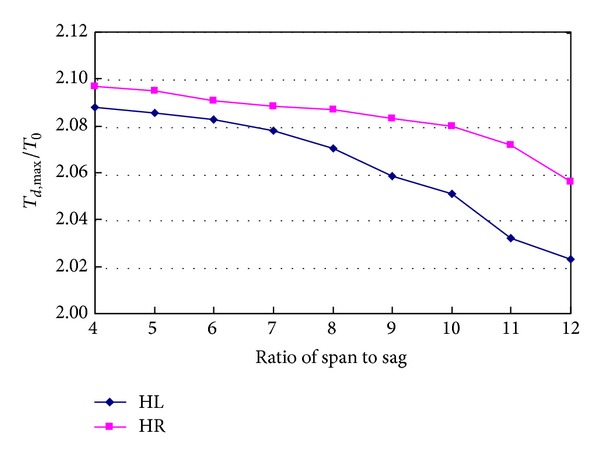
Effects of span to sag of main cable on hanger tension.

Span to sag ratio of main cable is a very important design parameter for suspension bridge, and it has large effects on the mechanic state of the bridge. When the span length of the bridge remains unchanged, the effects of span to sag ratio of main cable on the dynamic responses caused by sudden breakage of hanger H0 are studied.  Figure 19 shows the relationship between tension ratios of hanger and the ratio of span to sag of main cable. It can be seen from the figure that the tension ratio of hanger decreases with the ratio of span to sag increasing. When the span to sag ratio increases from 4 to 12, tension ratio of hanger HL decreases from 2.088 to 2.023, and tension ratio of hanger HR decreases from 2.097 to 2.056. They decrease by 3.1% and 2.0%, respectively.

### 4.6. Distance of Hanger

**Figure 20:**
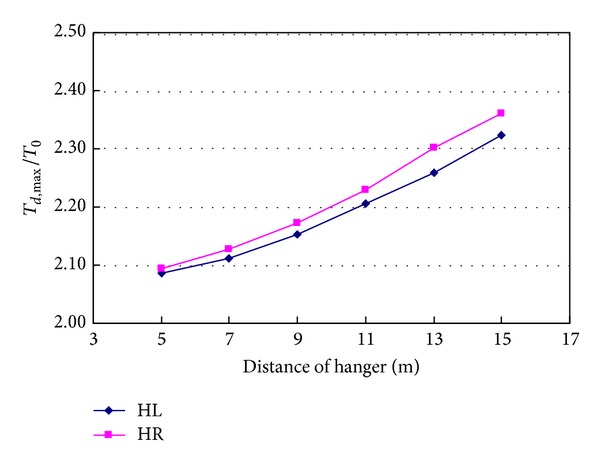
Effects of distance of hanger on hanger tension.

When the span length of the bridge remains unchanged, the effects of distance of hanger on the dynamic responses caused by sudden breakage of hanger H0 are studied.  Figure 20 shows the relationship between tension ratios of hanger and the distance of hanger. The tension ratio of hanger increases with the distance of hanger increasing. When the distance of hanger increases from 5 m to 15 m, tension ratio of hanger HL increases from 2.086 to 2.324, and tension ratio of hanger HR increases from 2.093 to 2.360. The tension ratios of the two hangers increase by 11.4% and 12.8%, respectively. So, the distance of hanger has significant effect on the dynamic responses caused by sudden breakage of a hanger.

### 4.7. Span Length

**Figure 21:**
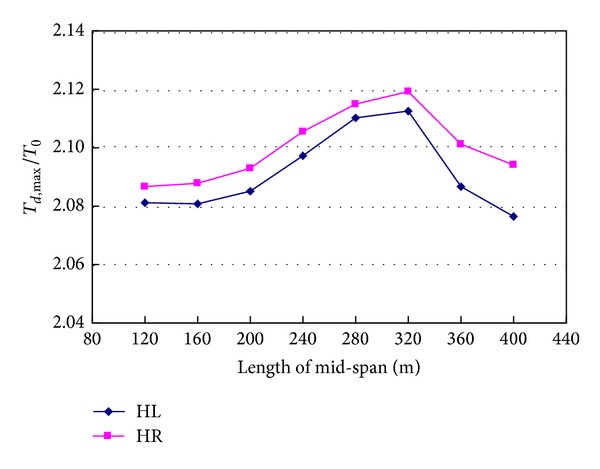
Effects of mid-span length on hanger tension.

The mid-span length of the actual bridge is 200 m. In order to study the effects of span length on the dynamic responses caused by sudden breakage of hanger, the mid-span length is changed from 120 m to 400 m. In the parametric study, the distances of hangers remain to be 5 m.  Figure 21 shows the relationship between tension ratios of hanger and mid-span length. With the mid-span length increasing, the tension ratio of hanger increases firstly and then decreases. The minimum value of tension ratio of hanger HL is 2.077, and the maximum value is 2.113, which is larger than the minimum value by 1.7%. The minimum value of tension ratio of hanger HR is 2.087, and the maximum value is 2.119, which is larger than the minimum value by 1.5%.

### 4.8. Breakage Time of Hanger

**Figure 22:**
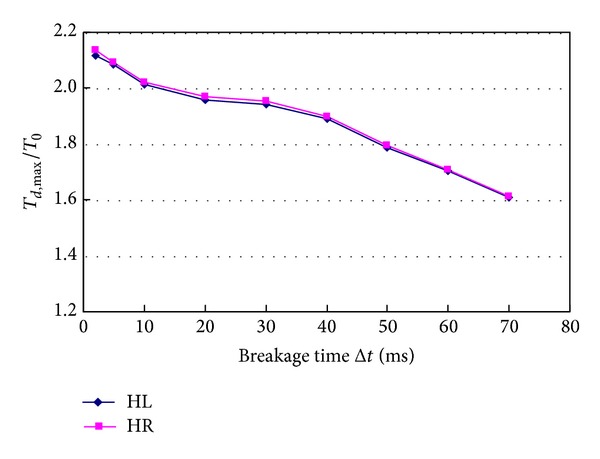
Effects of breakage time of hanger on hanger tension.

Figure 22 shows effects of breakage time* Δt* of hanger on hanger tension ratios. The breakage time of hanger affects the tension ratio of hanger very markedly. The tension ratio of hanger decreases rapidly with the breakage time increasing. When the breakage time increases from 2 ms to 70 ms, tension ratio of hanger HL decreases from 2.118 to 1.607, and tension ratio of hanger HR decreases from 2.135 to 1.612. The tension ratios of the two hangers increase by 24.1% and 24.5%, respectively. So, the breakage time of hanger should be studied in detail through experimental studies to make the numerical study be more accurate.

## 5. Conclusions

For self-anchored suspension bridge, its main cables are anchored directly to two ends of its stiffening girder, and the stiffening girder subjected to a very large compression force is supported by hangers. So the hangers are very important for the safety of the whole bridge. With the time increasing, the resistance capacities of the hangers decrease due to corrosion, and the hangers may break suddenly. The study on the responses of a self-anchored suspension bridge to breakage of hangers reaches the following conclusions.

1. The sudden breakage of a hanger produces a very strong vibration and large changes of internal forces of the bridge. During the vibration, the maximum tension of hanger reaches 2.22 times the initial value. The maximum increment of reaction force of bearing is 1.86 times the tension of the broken hanger, and the maximum decrement of reaction force of bearing is 1.54 times of tension of the broken hanger.
2. Within the studied influencing factors, the flexural stiffness of main cable, distance of hangers, and breakage time of hanger have very significant effects on the dynamic responses caused by sudden breakage of a hanger. Their effects on hanger tension ratio are larger than 10%. Torsion stiffness of girder, weight of main cable, span to sag ratio of main cable, and span length have significant effects on the dynamic responses caused by sudden breakage of a hanger, and their effects on hanger tension ratio are in the range of 2~5%. Flexural stiffness and weight of girder have very small effects on the dynamic responses caused by sudden breakage of hanger, and their effects on hanger tension ratio are less than 1%. Some of the above influencing factors have marked effects on reactions of the bearings. Particularly, when the factors take some values, the torsion vibrations of girder are very strong, and the bearing reactions occur to be very large values, which are several times of the tension of broken hanger.

## References

[1] Wang LL, Yi WJ (2007). Cases analysis on cable corrosion of cable-stayed bridges. *Central South Highway Engineering*.

[2] Ruiz-Teran AM, Aparicio AC (2007). Dynamic amplification factors in cable-stayed structures. *Journal of Sound and Vibration*.

[3] Wolff M, Starossek U Robustness assessment of a cable-stayed bridge.

[4] Wolff M, Starossek U Cable-loss analyses and collapse behavior of cable-stayed bridges.

[5] Mozos CM, Aparicio AC (2010). Parametric study on the dynamic response of cable stayed bridges to the sudden failure of a stay, Part I: bending moment acting on the deck. *Engineering Structures*.

[6] Mozos CM, Aparicio AC (2010). Parametric study on the dynamic response of cable stayed bridges to the sudden failure of a stay, Part II: bending moment acting on the pylons and stress on the stays. *Engineering Structures*.

[7] Kao CS, Kou CH (2010). The influence of broken cables on the structural behavior of long-span cable-stayed bridges. *Journal of Marine Science and Technology*.

[8] Zhou Y, Chen S (2014). Time-progressive dynamic assessment of abrupt cable-breakage events on cable-stayed bridges. *Journal of Bridge Engineering*.

[9] Post-Tensioning Institute (PTI) (2007). *Recommendations for Stay Cable Design*.

[10] Mozos CM, Aparicio AC (2011). Numerical and experimental study on the interaction cable structure during the failure of a stay in a cable stayed bridge. *Engineering Structures*.

[11] Cai JG, Xu YX, Zhuang LP, Feng J, Zhang J (2012). Comparison of various procedures for progressive collapse analysis of cable-stayed bridges. *Journal of Zhejiang University: Science A*.

[12] Qu ZL, Shi XF, Li XX, Ruan X (2009). Research on dynamic simulation methodology for cable loss of cable stayed bridges. *Structural Engineers*.

[13] Ruiz-Teran AM, Aparicio AC (2009). Response of under-deck cable-stayed bridges to the accidental breakage of stay cables. *Engineering Structures*.

[14] Qiu WL, Jiang M, Zhang Z (2009). Influencing factors of ultimate load carrying capacity of self-anchored concrete suspension bridge. *Journal of Harbin Institute of Technology*.

[15] Kao CS, Kou CH, Qiu WL, Tsai JL (2012). Ultimate load-bearing capacity of self-anchored suspension bridges. *Journal of Marine Science and Technology*.

[16] Zhang Z, Teng QJ, Qiu WL (2006). Recent concrete, self-anchored suspension bridges in China. *Proceedings of the Institution of Civil Engineers: Bridge Engineering*.

[17] Ministry of Transport of the People's Republic of China (2002). *Design Specifications for Highway Suspension Bridge*.

